# Progressive APOBEC3B mRNA expression in distant breast cancer metastases

**DOI:** 10.1371/journal.pone.0171343

**Published:** 2017-01-31

**Authors:** Anieta M. Sieuwerts, Willemijne A. M. E. Schrijver, Simone U. Dalm, Vanja de Weerd, Cathy B. Moelans, Natalie ter Hoeve, Paul J. van Diest, John W. M. Martens, Carolien H. M. van Deurzen

**Affiliations:** 1 Department of Medical Oncology, Erasmus MC Cancer Institute, Erasmus MC, Rotterdam, The Netherlands; 2 Cancer Genomics Center, Erasmus MC Cancer Institute, Erasmus MC, Rotterdam, The Netherlands; 3 Department of Pathology, University Medical Center Utrecht, Utrecht, The Netherlands; 4 Department of Radiology and Nuclear Medicine, Erasmus MC, Rotterdam, The Netherlands; 5 Department of Pathology, Erasmus MC, Rotterdam, The Netherlands; University of South Alabama Mitchell Cancer Institute, UNITED STATES

## Abstract

**Background:**

APOBEC3B was recently identified as a gain-of-function enzymatic source of mutagenesis, which may offer novel therapeutic options with molecules that specifically target this enzyme. In primary breast cancer, *APOBEC3B* mRNA is deregulated in a substantial proportion of cases and its expression is associated with poor prognosis. However, its expression in breast cancer metastases, which are the main causes of breast cancer-related death, remained to be elucidated.

**Patients and methods:**

RNA was isolated from 55 primary breast cancers and paired metastases, including regional lymph node (*N* = 20) and distant metastases (*N* = 35). *APOBEC3B* mRNA levels were measured by RT-qPCR. Expression levels of the primary tumors and corresponding metastases were compared, including subgroup analysis by estrogen receptor (ER/*ESR1*) status.

**Results:**

Overall, *APOBEC3B* mRNA levels of distant metastases were significantly higher as compared to the corresponding primary breast tumor (*P* = 0.0015), an effect that was not seen for loco-regional lymph node metastases (*P* = 0.23). Subgroup analysis by ER*-*status showed that increased *APOBEC3B* levels in distant metastases were restricted to metastases arising from ER-positive primary breast cancers (*P* = 0.002). However, regarding ER-negative primary tumors, only loco-regional lymph node metastases showed increased *APOBEC3B* expression when compared to the corresponding primary tumor (*P* = 0.028).

**Conclusion:**

*APOBEC3B* mRNA levels are significantly higher in breast cancer metastases as compared to the corresponding ER-positive primary tumors. This suggests a potential role for APOBEC3B in luminal breast cancer progression, and consequently, a promising role for anti-APOBEC3B therapies in advanced stages of this frequent form of breast cancer.

**Highlights**

- APOBEC3B is a gain-of-function enzymatic source of mutagenesis.
- Levels are higher in breast metastases as compared to corresponding primary tumors.
- This implies a novel role for APOBEC3B during breast cancer progression.
- This makes APOBEC3B a promising target for anti-APOBEC3B therapies.
- Especially in advanced stages of breast cancer.

## Introduction

Breast cancer is the fifth cause of overall cancer related death [1] and this mortality is largely caused by progression of metastatic disease [2]. Therefore, one of the most important challenges in breast cancer research includes the genetic changes and molecular mechanisms by which cancer cells acquire their metastatic ability. The generally accepted hypothesis is that metastases are caused by multiple intricate steps that arise in the primary tumor site [3]. Nevertheless, discordances between primary tumors and corresponding metastases are often encountered [4]. However, therapies applied for disseminated disease are mainly based on primary tumor characteristics only. The study of molecular differences between matched primary tumors and metastatic lesions may improve our understanding of disease progression and has the potential to reveal novel, potentially targetable drivers of metastatic progression.

Apolipoprotein B mRNA Editing Enzyme, Catalytic Polypeptide-Like 3B (APOBEC3B) is a member of the AID/APOBEC family of deaminases, which is recognized for its ability to deaminate genomic DNA cytosines. APOBEC enzymes normally function in the innate immune system and in the protection against viral pathogens, but these enzymes can also generate C→T mutations in the host genome [5]. Recently, several studies showed that APOBEC3B is a common enzymatic mutagenic factor affecting the evolution of different cancer types, including breast cancer [5–19].

In breast cancer, *APOBEC3B* mRNA is substantially upregulated in one third of cases and its expression is associated with mutational load, including certain driver mutations in *PIK3CA* and *TP53* [18,20]. Besides, multiple studies have postulated that APOBEC3B influences the development of metastases and drug resistance, especially in estrogen receptor alpha (ERα)-positive breast cancer [5,21,22]. In line with this, we previously reported an association between high *APOBEC3B* mRNA expression and poor outcome in a large cohort of patients with ERα-positive breast cancer [23].

Since APOBEC3B is a gain-of-function mutagenic enzyme, it may be treatable with small molecules [5,24], which could have an important role in the management of metastatic disease. However, the expression of *APOBEC3B* in breast cancer metastases remained to be elucidated.

In this study, we therefore quantified *APOBEC3B* mRNA in primary breast cancers and paired metastases to gain more insight into the levels of expression during breast cancer progression.

## Materials and methods

### Clinical pathological data

In this study we adhered to the Code of Conduct of the Federation of Medical Scientific Societies in the Netherlands (http://www.fmwv.nl) and the study making secondary use of human materials has been approved by our ‘Medische Ethnische Toetsing Commissie’ (METC; MEC 02.953). The use of anonymous or coded left over material for scientific purposes is part of the standard treatment agreement with patients and therefore informed consent was not required according to Dutch law [25]. We selected 73 formalin-fixed paraffin-embedded (FFPE) primary breast cancers and corresponding metastases from the pathology archives of the University Medical Center Utrecht and Erasmus Medical Center Rotterdam. Each specimen was reviewed by a pathologist to determine the percentage of invasive tumor cells. Inclusion criteria were: availability of clinical and pathological data, the presence of enough tumor tissue with the possibility to macro-dissect an area containing at least 50% tumor cells and good RNA quality and quantity to reliably determine expression levels by RT-qPCR (see also below). After applying these inclusion criteria, 55 paired primary tumors and metastases from different sites remained, including those from regional lymph nodes (*N* = 20), brain (*N* = 14), liver (*N* = 6), ovary (*N* = 4), lung (*N* = 4), bone (*N* = 4) and gastrointestinal tract (*N* = 3). Clinicopathological characteristics included age, primary tumor size, histological subtype, Bloom & Richardson score, ERα and human epidermal growth factor receptor 2 (HER2) expression and regional lymph node status. Furthermore, overall survival (death due to any cause) was reported. Detailed clinical information of this cohort is summarized in Table 1.

**Table 1:** Association of *APOBEC3B* mRNA expression with clinicopathological characteristics of the primary tumor.

			*APOBEC3B* mRNA log2		*AVG epithelial* mRNA log2		*PTPRC* (CD45) mRNA log2	
Clinical characteristics	No of patients*	Percentage of patients	Median	*IQR*	Median	*IQR*	Median	*IQR*^≠^
**All patients in this cohort**	55	100%	-6.14	*-5*.*28*	-3.11	*-1*.*25*	-2.98	*-2*.*31*
**Age at surgery (years)**								
≤ 40	10	18%	-6.69	*-4*.*62*	-2.74	*-1*.*40*	-2.31	*-2*.*89*
41–55	21	38%	-5.80	*-5*.*95*	-3.23	*-1*.*46*	-3.14	*-1*.*41*
56–70	20	36%	-6.64	*-4*.*16*	-3.03	*-1*.*25*	-3.48	*-7*.*68*
> 70	5	9%	-6.04	*-2*.*08*	-2.93	*-0*.*54*	-2.40	*-1*.*09*
*P*^≠^			*0*.*76*		*0*.*52*		*1*.*00*	
**Tumor size**								
≤ 2 cm	17	31%	-6.14	*-6*.*31*	-3.03	*-0*.*94*	-2.72	*-2*.*28*
2 ≤ 5 cm	27	49%	-5.92	*-2*.*25*	-2.93	*-1*.*40*	-3.62	*-7*.*65*
> 5 cm	7	13%	-6.91	*-4*.*07*	-3.17	*-1*.*19*	-3.27	*-1*.*69*
*P*^≠^			*0*.*70*		*0*.*95*		*0*.*88*	
**Histopathological subtypes**†								
Ductal	43	78%	-5.80	*-4*.*65*	-2.93	*-1*.*41*	-3.27	*-1*.*76*
Lobular	8	15%	-8.50	*-3*.*92*	-3.14	*-0*.*85*	-2.31	*-4*.*90*
Other	4	7%	-6.43	*-3*.*19*	-3.37	*-0*.*58*	-1.23	*-2*.*24*
*P*^$^			*0*.*07*		*0*.*41*		*0*.*31*	
**Bloom & Richardson grade**								
I + II	10	18%	-6.39	*-4*.*78*	-3.11	*-1*.*51*	-6.94	*-7*.*56*
III	38	69%	-5.78	*-4*.*65*	-3.15	*-1*.*33*	-2.92	*-2*.*37*
*P*^$^			*0*.*43*		*0*.*52*		*0*.*08*	
***ESR1* status**								
Negative	22	40%	-5.64	*-3*.*79*	-2.91	*-1*.*24*	-3.06	*-2*.*00*
Positive	33	60%	-6.40	*-4*.*69*	-3.17	*-1*.*35*	-2.98	*-2*.*81*
*P*^$^			*0*.*15*		*0*.*39*		*0*.*88*	
***ERBB2* status**								
Negative	43	78%	-5.80	*-5*.*01*	-3.19	*-1*.*06*	-2.97	*-2*.*90*
Positive/amplified	12	22%	-7.35	*-3*.*70*	-2.77	*-1*.*59*	-3.31	*-1*.*67*
*P*^$^			***0*.*04***		*0*.*11*		*0*.*96*	
**Regional lymph node status**								
Negative	16	29%	-5.64	*-4*.*40*	-3.17	*-1*.*14*	-2.84	*-2*.*63*
Positive	33	60%	-6.40	*-4*.*62*	-3.00	*-1*.*17*	-2.98	*-1*.*84*
*P*^$^			*0*.*23*		*0*.*74*		*0*.*90*	
**Time between primary tumor and studied metastasis**								
≤ 24 months	33	60%	-5.92	*-5*.*45*	-2.88	*-1*.*16*	-2.85	*-2*.*09*
> 24 months	22	40%	-6.43	*-4*.*09*	-3.24	*-0*.*93*	-3.20	*-2*.*28*
*P*^≠^			0.97		*0*.*42*		*0*.*33*	
**Overall survival status**								
Alive	22	40%	-5.92	*-2*.*52*	-3.11	*-1*.*48*	-3.67	*-8*.*34*
Deceased	33	60%	-6.65	*-5*.*03*	-3.11	*-0*.*95*	-2.81	*-1*.*68*
*P*^$^			*0*.*21*		*0*.*62*		*0*.*20*	

AVG epithelial; average mRNA level of *KRT19* and *EPCAM*. IQR; interquartile range.

* Due to missing values numbers do not always add up to 55.

^*≠*^ Spearman correlation significance (2-tailed).

^$^ Mann-Whitney Test significance (2-tailed).

^†^ mRNA expression of ductal and lobular breast cancer was compared.

### RNA isolation and quantitative reverse transcriptase polymerase chain reaction (RT-qPCR)

Ten 10 μm slides were cut from the primary tumors and paired metastases. The first and last sections (5 μm) were stained with hematoxylin and eosin to guide macro-dissection of the tumor cells for RNA extraction. Total RNA was isolated from the macro-dissected sections with the AllPrep DNA/RNA FFPE Kit (Qiagen) and resulting nucleic acid concentrations were measured with a Nanodrop 2000 system (ThermoFisher Scientific). cDNA was generated for 30 min at 48°C with RevertAid H minus (ThermoFisher Scientific) and gene-specific pre-amplified with Taqman PreAmp Master mix (ThermoFisher Scientific) for 15 cycles, followed by Taqman probe—based real time PCR according to the manufacturer’s instructions in a MX3000P Real-Time PCR System (Agilent). The following gene expression assays were evaluated (all from ThermoFisher Scientific): *APOBEC3B*, Hs00358981_m1; *EPCAM*, Hs00158980_m1; *ESR1*, Hs00174860_m1; *ERBB2*, Hs01001580_m1, *KRT19*, Hs01051611_gH; *PTPRC*, Hs00236304_m1. mRNA levels were quantified relative to the average expression of *GUSB*, Hs9999908_m1 and *HMBS*, Hs00609297_m1 using the delta Cq (dCq = 2^(average Cq reference genes—Cq target gene)) method.

### Quality and quantity control measurements for reliable quantitative reverse transcriptase polymerase chain reaction (RT-qPCR)

For reliable RT-qPCR measurements, only samples that resulted in amplifiable products within 25 cycles for the used reference gene set at an input of 50 ng total RNA (92.9% of the samples) were considered to be of good quality to reliably determine RT-qPCR levels. Furthermore, a serially diluted FFPE breast tumor sample was included in each experiment to evaluate the linear amplification and efficiencies for all genes included in the panel and absence of amplification in the absence of reverse transcriptase. All gene transcripts were 100% efficient amplified (range 94%-102%) and were negative in the absence of reverse transcriptase.

### Estrogen receptor (ER/*ESR1*) and HER2 (HER2/*ERBB2*) status

**Fig 1:**
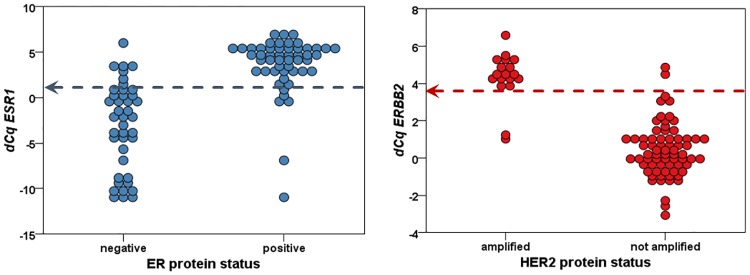
Correlation of ER and HER2 protein status with *ESR1* and *ERBB2* mRNA levels. Arrows indicate used cut-off value.

Because data regarding ER and HER2 expression on protein level of our data set was incomplete, *ESR1* and *ERBB2* mRNA expression was used to determine *ESR1* and *ERBB2* mRNA status (using a cut-off dCq for *ESR1*>1 and *ERBB2*>3.5 by optimal binning for n = 92 and n = 87 overlapping samples, respectively (Fig 1)). Because ER and HER2 are determined on protein level in daily clinical practice (using a scoring system according to national guidelines [26,27]), we investigated whether the *ESR1* and *ERBB2* mRNA status accurately reflected the ER and HER2 protein status as reported in the pathology reports in samples with known receptor protein status (Fig 1). These cut-offs resulted in a sensitivity of 0.88 and specificity of 0.85 for *ESR1* and in a sensitivity of 0.89 and specificity of 0.97 for *ERBB2*.

### Statistics

SPSS version 23 was used for all statistical analyses. The Kolmogorov-Smirnov and Shapiro-Wilk tests were used to test for normality of the distributions. To compare mean values between two or more groups, the Mann-Whitney U Test or Kruskal-Wallis test was used, followed by a test for trend if appropriate. To compare values measured in primary cancers and paired metastases, the paired Wilcoxon Signed Ranks test was used. To correlate linear variables, the Spearman Rank Correlation test was used. *P*-values ≤ 0.05 were considered statistically significant.

## Results

### *APOBEC3B* mRNA expression in primary breast cancer

Since the Kolmogorov-Smirnov and Shapiro-Wilk tests showed that our data were not always normally distributed, we tested all our data non-parametrically. First, we correlated the levels of *APOBEC3B* mRNA measured in the primary tumors with traditional clinicopathological characteristics (Table 1). Besides a higher expression of *APOBEC3B* mRNA in *ERBB2* negative tumors when compared to *ERBB2* positive tumors (Spearman’s Rho = -0.46, *P* = 0.002), *APOBEC3B* mRNA levels were not correlated to any of the studied parameters.

To ensure that different levels of tumor cells or inflammatory cells in the primary tumors and their matched metastasis did not bias our data, we quantified the mRNA levels of *KRT19* and *EPCAM* (as a measure for epithelial content) and *PTPRC (*the gene for the common leukocyte antigen CD45, as a measure for the presence of lymphocytes). Although a weak positive correlation was observed between *APOBEC3B* mRNA levels and epithelial content in the complete cohort (Spearman’s Rho = 0.21, *P* = 0.031, *N* = 110), no significant correlations were observed between the mRNA levels of *APOBEC3B* and epithelial or infiltrate content when analyzed separately for the primary tumors and the metastases (Spearman correlation significance *P* > 0.05). In addition, epithelial and infiltrate content did not differ significantly between the primary tumors and matched metastases (paired Wilcoxon Signed Rank test *P* > 0.05).

### *APOBEC3B* mRNA expression in primary breast cancer and paired metastases

**Fig 2:**
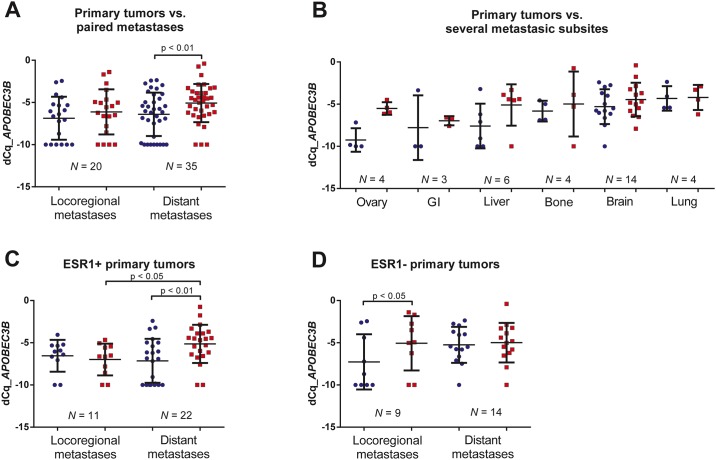
*APOBEC3B* mRNA expression differences between primary breast tumors and paired metastases. *(A) APOBEC3B* mRNA expression in primary breast tumors versus paired loco-regional and distant metastases. *(B) APOBEC3B* mRNA expression in primary breast tumors versus paired metastases, subdivided per location of metastasis (ovary (N = 4); liver (N = 6); bone (N = 4); brain (N = 14); lung (N = 5) and gastro-intestinal tract (N = 3). *(C) APOBEC3B* mRNA expression in *ESR1*-positive primary breast tumors versus paired distant and loco-regional metastases. *(D) APOBEC3B* mRNA expression in *ESR1*-negative primary breast tumors versus paired distant and loco-regional metastases. P-values obtained by paired Wilcoxon Signed Ranks test (2-tailed).

Next, we correlated *APOBEC3B* mRNA expression in primary tumors and their matched metastases. This analysis revealed that *APOBEC3B* mRNA levels were significantly higher in the matched distant metastases as compared to the primary tumors (paired Wilcoxon Signed Rank test *P* = 0.0015, Fig 2A and Table 2). In contrast, no difference was perceived between primary tumors and matched loco-regional lymph node metastases (paired Wilcoxon Signed Rank test *P* = 0.23)), while levels remained significantly elevated for the cohort with distant metastases (paired Wilcoxon Signed Rank test P = 0.02); (Table 2). Also, *APOBEC3B* mRNA levels measured in distant metastases (*N* = 35) showed a trend towards higher expression when compared to regional lymph node metastases (*N* = 20) of unmatched cases (Mann-Whitney U test *P* = 0.08, Fig 2A). No such trend was observed in primary tumors that disseminated either to loco-regional or distant locations (Mann-Whitney U test *P* = 0.42, Fig 2A).

**Table 2:** Association of *APOBEC3B* mRNA in matched primary tumors and their metastasis according metastatic site.

			*APOBEC3B* mRNA log2		*AVG epithelial* mRNA log2		*PTPRC* (CD45) mRNA log2	
Clinical parameter	No of patients*	Percentage of patients	Mean	*SD*	Mean	*SD*	Mean	*SD*
**Tissue origin**								
Primary tumor	55	100%	-6.51	*2*.*53*	-3.04	*1*.*07*	-3.98	*3*.*23*
Paired Metastasis	55	100%	-5.36	*2*.*39*	-2.85	*1*.*08*	-4.48	*2*.*84*
*P*^ǂ^			***0*.*0015***		*0*.*20*		*0*.*26*	
**According metastatic site**								
**Loco-regional lymph node**								
Primary tumor	20	36%	-6.87	*2*.*55*	-2.82	*0*.*91*	-3.88	*2*.*89*
Paired Metastasis	20	36%	-6.12	*2*.*68*	-2.63	*0*.*89*	-3.42	*3*.*03*
*P*^ǂ^			*0*.*23*		*0*.*31*		*0*.*49*	
**Distant metastases**								
Primary tumor	35	64%	-6.30	*2*.*54*	-3.16	*1*.*15*	-4.30	*3*.*34*
Paired Metastasis	35	64%	-4.93	*2*.*13*	-2.98	*1*.*16*	-4.82	*2*.*80*
*P*^ǂ^			***0*.*002***		*0*.*38*		*0*.*39*	
**Distant metastasis specified**								
**Ovary**								
Primary tumor	4	7%	-9.25	*1*.*39*	-3.31	*1*.*25*	-5.53	*5*.*25*
Paired Metastasis	4	7%	-5.51	*0*.*74*	-3.03	*1*.*20*	-3.99	*0*.*93*
*P*^ǂ^			*0*.*07*		*0*.*72*		*0*.*47*	
**GI tract**								
Primary tumor	3	5%	-7.79	*3*.*84*	-3.32	*0*.*20*	-1.83	*1*.*10*
Paired Metastasis	3	5%	-6.96	*0*.*54*	-3.93	*1*.*88*	-4.41	*4*.*88*
*P*^ǂ^			*0*.*59*		*0*.*59*		*0*.*11*	
**Liver**								
Primary tumor	6	11%	-7.59	*2*.*65*	-3.16	*1*.*57*	-2.66	*1*.*35*
Paired Metastasis	6	11%	-5.10	*2*.*45*	-2.20	*1*.*17*	-4.76	*2*.*71*
*P*^ǂ^			*0*.*08*		*0*.*17*		*0*.*028*	
**Bone**								
Primary tumor	4	7%	-5.82	*1*.*23*	-3.01	*1*.*28*	-5.04	*3*.*31*
Paired Metastasis	4	7%	-4.98	*3*.*85*	-2.63	*0*.*96*	-4.11	*3*.*97*
*P*^ǂ^			*0*.*47*		*0*.*27*		*0*.*47*	
**Brain**								
Primary tumor	14	25%	-5.29	*2*.*06*	-3.10	*1*.*03*	-5.53	*3*.*58*
Paired Metastasis	14	25%	-4.46	*2*.*00*	-2.95	*0*.*81*	-5.67	*2*.*82*
*P*^ǂ^			*0*.*20*		*0*.*68*		*0*.*98*	
**Lung**								
Primary tumor	4	7%	-4.32	*1*.*45*	-3.29	*1*.*69*	-2.35	*1*.*59*
Paired Metastasis	4	7%	-4.21	*1*.*49*	-3.82	*1*.*47*	-3.76	*1*.*66*
*P*^ǂ^			*0*.*72*		*0*.*47*		*0*.*14*	

SD; standard deviation. AVG epithelial; average mRNA level of *KRT19* and *EPCAM*.

* Due to missing values, numbers don’t always add up to 55.

^ǂ^ Paired Wilcoxon Signed Ranks Test significance (2-tailed).

Subgroup analysis by distant metastatic site showed increased *APOBEC3B* expression for all locations, particularly for liver and ovary, although no significance was reached for any of the relatively small subgroups (paired Wilcoxon Signed Ranks test *P* > 0.05, Fig 2B and Table 2). We also compared mRNA levels measured in primary tumors according to distant metastatic site. These analyses showed that *APOBEC3B* mRNA levels were lowest in primary tumors that metastasized to the ovaries and gastro-intestinal sites and highest in primary tumors that metastasized to lung, brain or bone (Kruskal-Wallis test *P* = 0.030), Fig 2B).

### *APOBEC3B* mRNA expression according to *ESR1* and *ERBB2* status of the primary tumor

We observed no association between *APOBEC3B* mRNA levels and *ESR1*-status (Table 1). Several previous studies, however, showed higher *APOBEC3B* mRNA expression in ERα-negative tumors compared to ERα-positive tumors [23,28,29]. Notably, in these studies, high *APOBEC3B* expression levels were only associated with poor prognosis for ERα-positive primary breast tumors. We therefore categorized our primary cohort into *ESR1*-positive and *ESR1*-negative primary tumors (Fig 2C and 2D). For the *ESR1*-positive primary tumors, a significantly higher expression was seen in paired distant metastases, but not in loco-regional metastases (Fig 2C; paired Wilcoxon Signed Ranks Test *P* = 0.002 and 0.53, respectively). In contrast, for the *ESR1*-negative primary tumors a significantly higher expression was seen in loco-regional metastases, but not in distant metastases (Fig 2D; paired Wilcoxon Signed Ranks Test *P* = 0.028 and 0.81, respectively). Receptor conversion from an *ESR1*-positive primary tumor to an *ESR1*-negative metastasis could not explain this finding (Table 3).

**Table 3:** *ESR1* conversions from primary tumor to metastasis specified by site of metastasis.

*ESR1* conversion primary to metastasis	Metastasis type	*N*	*dCq APOBEC3B*	*dCq APOBEC3B*	*P*-value*	*P*-value*
			Primary (Average)	Metastasis (Average)		
Not converted	loco-regional lymph node	15	-6.4	-5.97	0.61	**0.032**
	distant metastasis	27	-5.86	-4.87	0.015	
*ESR1-* primary to *ESR1+*metastasis	loco-regional lymph node	5	-8.26	-6.57	0.11	**0.041**
*ESR1+* primary to *ESR1-* metastasis	distant metastasis	8	-7.8	-5.16	0.07	

* Wilcoxon Signed Ranks Test

No such difference was seen after categorizing our patients according to *ERBB2*-status. Irrespective of *ERBB2*-status, *APOBEC3B* levels were only higher in the distant metastases and not in the loco-regional lymph nodes when compared to the paired primary tumor (paired Wilcoxon Signed Ranks Test *P* < 0.05 and > 0.05, respectively).

## Discussion

APOBEC3B is thought to affect the evolution of breast cancer by somatically mutagenizing the cancer genome, which could potentially be abrogated by therapeutic intervention [5]. Previous studies investigated *APOBEC3B* mRNA expression in primary breast tumors and paired normal tissue. These studies reported upregulation in primary tumors compared to normal tissue, especially in ERα-negative cases [17,28]. However, metastatic disease remains the major cause of breast cancer related mortality and several studies reported discordances of (epi) genetic and immunohistochemical markers between primary tumors and matched metastases [4,30–34]. To the best of our knowledge, no data is available regarding APOBEC3B expression in breast cancer metastases. Therefore, we set out to evaluate *APOBEC3B* mRNA expression in primary breast cancer and matched metastases. Importantly, we encountered a significant increase in *APOBEC3B* mRNA levels in the metastases compared to their corresponding primary tumor. Furthermore, distant metastases showed higher expression than loco-regional lymph node metastases. This implies a role for APOBEC3B not only at the stage of the primary tumor but also, and according to our data even more dominantly, during tumor evolution of metastatic breast cancer.

Previous studies reported an association between *APOBEC3B* expression and aggressive characteristics of the primary breast cancer, including high histologic grade, genomic grade, advanced stage, negative ERα status and HER2/*ERBB2* amplification [21,23,28,29,35]. In this current, more concise study with a special focus on breast cancer metastases, we only observed a negative association between *APOBEC3B* and *ERBB2* mRNA levels in both the primary tumor (Table 1) and the metastases (data not shown). However, our sample size was relatively small with a relatively high number of cases with loco-regional (36%) and brain metastases (25%), which could have biased our results.

Overall, we did not find a correlation between *APOBEC3B* and *ESR1*-status of the primary tumor, while previous studies reported higher *APOBEC3B* mRNA expression in ERα-negative tumors compared to ERα-positive tumors ([23,28,29]. Interestingly, for our *ESR1*-negative primary cases, a significantly higher expression of *APOBEC3B* was seen in paired loco-regional metastases only and not in paired distant metastases. For our *ESR1*-positive primary cases on the other hand, a significantly higher expression was seen in paired distant metastases and not in the loco-regional lymph nodes. This is especially noteworthy in view of our previous finding, that high levels of *APOBEC3B* were only associated with poor prognosis in *ESR1*-positive primary breast cancers, and not in *ESR1*-negative cases [23].

Irrespective of *ESR1*-status, we showed that *APOBEC3B* expression was increased in distant metastases compared to the corresponding primary tumor, with highest expression in liver, lung, brain and bone metastases. APOBEC3B thus seems not only needed for breast cancer progression, but also for maintenance of the metastasis in distant environments. Since APOBEC3B is upregulated in numerous cancer types we wondered if these findings could be explained by the micro-environment of the distant site. In an article of Burns et al. [7], *APOBEC3B* expression levels determined by RNA-seq showed a lower expression in normal brain and ovarian tissue relative to normal breast tissue. Furthermore, brain tumors (low-grade glioma, glioblastoma multiforme) and ovarian tumors (serous cystadenocarcinoma) also had lower *APOBEC3B* expression levels than breast carcinoma. This might imply that the higher *APOBEC3B* mRNA levels we found in breast cancer brain and ovarian metastases are independent of the micro-environment at these locations. Furthermore, since the pattern of *APOBEC3B* expression in primary tumors is retained and even increased in paired metastases, and shows a trend toward a possible metastatic location-specific pattern, one could envision that the primary tumor is already ‘primed’ for an eventual site of dissemination. This should however be validated in a larger cohort and also at the protein level. To this end, we tried 2 commercially available APOBEC3B antibodies (PAB2474 from Abnova and Anti-APOBEC3B antibody—N-terminal ab191695 from Abcam). However, despite various efforts, we had to conclude that detecting APOBEC3B in breast cancer by immunohistochemistry with these currently commercially available antibodies should be considered unreliable due to non-specific staining. Hopefully, now that APOBEC3B is gaining increased interest, more specific antibodies will become available soon to confirm our findings at the protein level.

In theory, tumor heterogeneity could explain some of the observed differences between primary tumors and paired metastases. However, the reported distinct *APOBEC3B* mRNA expression levels of the primary tumor that were largely retained or increased in the paired metastases could not solely be explained by heterogeneity. In daily practice, the majority of metastases are not resected or biopsied. This likely resulted in a selection bias, since we only included primary tumors with available material of the paired metastasis. Another weakness of our study is the relatively small number of patients with distant metastases, which limited the reliability of subgroup analysis according to metastatic site.

In conclusion, our findings add to the knowledge that *APOBEC3B* contributes to breast cancer progression and has now extended this to metastatic disease. Since *APOBEC3B* expression is at least retained and often even increased in distant metastases, our data suggest that it might also be an effective interventional candidate for disseminated breast cancer.

## Abbreviations

APOBEC3B: Apolipoprotein B mRNA Editing Enzyme, Catalytic Polypeptide-Like 3B
AVG: average
Cq: quantitative threshold cycle
dCq: delta quantitative threshold cycle
ER/ESR1: estrogen receptor
ERα: estrogen receptor alpha
FFPE: formalin-fixed paraffin-embedded
HER2/ERBB2: human epidermal growth factor receptor 2
NWO: Netherlands Organization of Scientific Research
PTPRC: gene for the common leukocyte antigen CD45
RT-qPCR: quantitative reverse transcriptase polymerase chain reaction
